# Health Behaviour and Body Esteem Profiles in Women Who Achieved a Normal BMI After Non-Pharmacological Weight Loss: Differences in Life Satisfaction

**DOI:** 10.3390/jcm15197690

**Published:** 2026-10-04

**Authors:** Klaudia Boniecka, Hanna Liberska

**Affiliations:** 1 Institute of Pedagogy and Psychology, Pomeranian University in Słupsk, ul. Arciszewskiego 22A, 76-200 Słupsk, Poland; 2 Faculty of Psychology, Kazimierz Wielki University, ul. Chodkiewicza 30, 85-064 Bydgoszcz, Poland; hanna.liberska@op.pl; 3 Institute of Psychology, University of Zielona Góra, ul. Licealna 9, 65-417 Zielona Góra, Poland

**Keywords:** non-pharmacological interventions, physical activity, weight loss, health behaviour profiles, health behaviours, body esteem, life satisfaction, health education

## Abstract

**Background/Objectives:** Women who reach a normal BMI after non-pharmacological weight loss may differ in health behaviours and body esteem. We identified profiles based on four health behaviour and three body esteem dimensions and compared their life satisfaction. **Methods:** This exploratory analysis included 122 women aged 20–37 who reported reaching a BMI of 18.5–24.9 kg/m^2^ and completed the second assessment of a two-stage study. They were married or cohabiting and had at least one child. Approximately 1000 women participated in the first assessment but were not actively followed. Standardised post-weight-loss indicators were analysed using Ward clustering and k-means++; solutions (k = 2–6) and stability were assessed using silhouette coefficients, classification agreement, and bootstrapping. **Results:** Two descriptive profiles emerged: relatively more favourable (*n* = 69) and relatively less favourable (n = 53). Overall life satisfaction was higher in the former (245.75 ± 46.63 vs. 197.77 ± 43.16; Hedges’ g = 1.06, 95% CI 0.68–1.44), as were scores across ten life domains. Profile membership was associated with the self-selected weight-loss method (χ^2^(3) = 53.80; *p* < 0.001; Cramer’s V = 0.66), without implying a causal advantage or effectiveness of any method. **Conclusions:** Among these 122 women, a similar final BMI co-occurred with distinct health behaviour and body esteem patterns associated with life satisfaction. Because participants who did not return were not actively followed, the frequency of these profiles among all initially assessed women or all women attempting weight loss cannot be estimated. The exploratory profiles require independent replication.

## 1. Introduction

Obesity is a chronic disease, and its management is long-term, multifactorial and complex with biological, psychological and social determinants [1,2]. Non-pharmacological interventions, including dietary changes, increased physical activity, self-control, and behavioural modification, remain a fundamental element of treating excess body weight and maintaining the achieved results [2,3,4]. However, their effectiveness is still most often assessed based on changes in body weight or BMI, even though these indicators do not describe the full clinical or psychosocial outcome [5].

In the present study, a favourable anthropometric outcome was operationalised as a reduction in BMI from at least 25 kg/m^2^ to 18.5–24.9 kg/m^2^. Focusing on women who reached this predefined endpoint was intentional: the present analysis does not evaluate weight-loss effectiveness but asks whether women with a similar final anthropometric status nevertheless show heterogeneous patterns of functioning. Restricting the analysis to a common BMI endpoint reduces variation attributable to final weight status and allows the analysis to focus on heterogeneity beyond BMI. This criterion does not, however, establish the durability of weight loss or imply that all women who begin weight reduction will achieve the same outcome [3,6,7,8].

Health-related behaviours are significant following weight loss, as they encompass daily patterns regarding nutrition, preventive care, health practices, and mindset that may persist after the anthropometric goal has been reached. Physical activity is a component of certain health practices and weight-loss strategies; however, in observational studies, its contribution cannot be disentangled from diet, motivation, support, and other factors [8].

Body esteem is a complementary psychological dimension of functioning after weight loss. Losing excess weight can improve body image and body esteem, but the magnitude of change varies and does not always correspond to the amount of weight lost [9,10]. Achieving a BMI within the normal range therefore does not necessarily imply comparable satisfaction with sexual attractiveness, weight-related aspects of the body, or physical condition [11,12,13].

From a biopsychosocial perspective, an anthropometric endpoint alone does not fully describe an individual’s state following weight loss. A person-centred approach makes it possible to examine whether various characteristics co-occur in individuals in distinct configurations, thereby complementing variable-centred analyses that focus on average associations. In the present study, health behaviours and body esteem were selected as profile indicators because, together, they represent the behavioural and psychological—body-related—dimensions of functioning after weight loss. It should be emphasised that the identified profiles constitute exploratory descriptive patterns rather than clinical categories.

Life satisfaction was treated as an external variable and was not included in the construction of the profiles. The self-selected weight loss method was analysed as an additional, secondary characteristic of profile membership rather than as an experimentally tested intervention.

The main objectives were: (1) to identify post-weight-loss profiles based on health behaviours and body esteem among women who achieved a BMI of 18.5–24.9 kg/m^2^ and (2) to compare these profiles in terms of overall life satisfaction and ten life domains. An exploratory objective was to examine the association between profile membership and the self-selected weight loss method.

## 2. Materials and Methods

### 2.1. Study Design and Participants

The original study employed a two-stage longitudinal design. The first assessment took place 1–7 days before the participant began a self-selected weight-loss method, and the second occurred 7–14 days after she reported achieving a BMI of 18.5–24.9 kg/m^2^. The present article is an exploratory profile analysis focusing on the post-weight-loss assessment; thus, regarding profile construction, it is cross-sectional in nature, even though it is embedded within a broader longitudinal project. An earlier publication from the original study [13] addressed longitudinal changes in psychological well-being and body image. The current analysis addresses a distinct research question using a person-centred approach, with health behaviours and body esteem as profile indicators and life satisfaction as an external variable. Data collected using the IZZ, BES, and FLZ at baseline and follow-up were used to determine whether the post-reduction profiles were preceded by differences in the same constructs (variables) prior to weight loss. The median duration of the weight loss process was 13 months (IQR 11.25–24; range 1–36 months). This duration did not differ significantly between the two profiles (medians 14 vs. 12 months; U = 2133; *p* = 0.114) or among the four categories of self-selected methods (H(3) = 4.69; *p* = 0.196).

The first assessment of the broader project involved about 1000 women. Purposive sampling was employed. The study was anonymous; each participant was assigned a non-identifying research number, and the two corresponding questionnaire packages—intended for the first and second assessments—were marked with the same number, enabling the linkage of results without the collection of personal data. Following the first assessment, participants were not actively monitored on an individual basis. The broader project was not designed as a study of participant attrition or as an evaluation of weight loss efficacy across the entire initial cohort. The current analysis focused on women who achieved a pre-defined BMI endpoint. Women who wished to continue participation and reported reaching the target BMI range returned for the second assessment. The analytical sample comprised 122 women aged 20–37 years (M = 29.16; SD = 4.17) who completed both assessments, met the inclusion criteria, and provided the complete dataset required for analysis. The mean initial BMI was 29.94 kg/m^2^ (SD = 3.72), and the final BMI was 22.35 kg/m^2^ (SD = 1.90); mean body weight decreased from 85.83 kg (SD = 12.62) to 64.00 kg (SD = 6.86).

Due to the lack of active follow-up for all participants from the initial group—including those who did not present for the second stage of the longitudinal study—it is not possible to reliably classify them retrospectively as individuals who failed to reach the BMI endpoint, discontinued weight loss, or achieved their goal but did not return for the second measurement. Inclusion criteria included female gender, adult status, an initial BMI of ≥25 kg/m^2^, a BMI of 18.5–24.9 kg/m^2^ at the time of the study, being in a marriage or cohabiting relationship, and having at least one child. The criteria regarding relationship status and parenthood stemmed from the broader parent study, which also examined partner and family functioning; these factors are not the focus of the current profile analysis and limit the generalisability of the findings. Women were excluded if their excess body weight was attributable to diagnosed hormonal or genetic disorders, central nervous system damage, or medication use. Body weight and height were self-reported by participants and were not subject to independent anthropometric verification. Questionnaires were completed anonymously, and incomplete sets were excluded prior to the formation of the final analytical sample.

### 2.2. Self-Selected Methods of Weight Loss

Participants independently selected their weight loss method. The study was observational and non-experimental; no weight loss method was assigned or modified. Four categories were identified in the source procedure: (1) dietary therapy and physical activity under the supervision of a dietitian (n = 30); (2) self-managed dietary therapy and physical activity (n = 32); (3) elimination diets (n = 28); and (4) low-calorie diets (n = 32). In the source questionnaire, low-calorie diets were described as strategies with significantly reduced caloric intake (<1200 kcal; examples included a 1000 kcal diet or fasting), whereas elimination diets were defined as strategies based on the significant restriction or exclusion of food groups or macronutrients (examples: Dukan, Kwaśniewski, carnivore, and paleo diets). Classification was based on the participant’s self-reported primary method. Adherence to recommendations and changes in method during the weight loss process were not measured in the current analysis. Therefore, associations between the method category and profile membership were interpreted as observational co-occurrences rather than evidence of efficacy or causality.

### 2.3. Tools

#### 2.3.1. Health Behaviour Inventory (IZZ) Z. Juczyński (2001) [14]

Juczyński’s Health Behaviour Inventory (IZZ) [14] includes 24 items concerning various types of health-related behaviours. Respondents respond to statements using a 5-point scale: from 1 (almost never) to 5 (almost always). The higher the total score, the greater the intensity of the declared health behaviours. The IZZ allows for determining the overall intensity of health behaviours and the intensity of four categories: healthy eating habits, preventive behaviours, health practices, and a positive mental attitude. Healthy eating habits refer to the type of food consumed; preventive behaviours refer to compliance with health recommendations and obtaining information about health and disease; health practices include habits related to sleep, physical activity, and recreation; and a positive mental attitude refers to avoiding excessive emotions, stress, tension, and depressing situations. The internal consistency of the Inventory, determined based on Cronbach’s alpha, is 0.85 for the overall health behaviour index, and for the individual subscales it ranges from 0.60 to 0.65. In a test–retest study with a six-week interval, a correlation coefficient of 0.88 was obtained. Validity was assessed by comparing the results obtained in the Inventory with the results of the Generalized Self-Efficacy Scale, the Life Orientation Test, and the Multidimensional Health Locus of Control Scale. In the present analytic sample, Cronbach’s alpha was 0.91 for the IZZ total score and ranged from 0.66 to 0.83 across the four subscales.

#### 2.3.2. Body Esteem Scale S.L. Franzoi and S.A. Shields (1984) [15], Polish Adaptation: M. Lipowska and M. Lipowski (2013) [16]

The Franzoi and Shields Body Esteem Scale [15], adapted in Polish by Lipowska and Lipowski [16], has 35 items arranged in three subscales, each for men and women. In women, the subscales measure sexual attractiveness, weight concern, and physical condition, while in men, they measure physical attractiveness, upper body strength, and physical condition. For women, sexual attractiveness refers to the assessment of appearance and aspects related to sexuality. Weight concern refers to the attitude towards one’s appetite and body parts that can be influenced by diet and physical activity. The physical condition subscale includes the assessment of body aspects related to endurance, strength, and agility. The participant rates body parts or body functions on a 5-point Likert scale, where 1 indicates extreme dissatisfaction, 3 indicates neutrality, and 5 indicates extreme satisfaction. The score for each subscale is calculated by summing the points; higher scores indicate greater body satisfaction in a given area. The reliability of the original version of the scale for women, depending on the subscale, ranged from 0.78 to 0.87 [15]. In the Polish version, Cronbach’s alpha coefficients were 0.80 for sexual attractiveness, 0.89 for weight concern, and 0.82 for physical condition [16]. In the present analytic sample, Cronbach’s alpha coefficients for the three Body Esteem subscales ranged from 0.82 to 0.88.

#### 2.3.3. Life Satisfaction Questionnaire (Fragebogen Zur Lebenszufriedenheit—FLZ) J. Fahrenberg, M. Myrtek, J. Schumacher and E. Brähler (2000) [17], Polish Adaptation: J. Chodkiewicz (2009) [18]

The FLZ Life Satisfaction Questionnaire [17], adapted in Polish by Chodkiewicz [18], was used to measure life satisfaction. The questionnaire assesses satisfaction with aspects of life such as health, work and profession, financial situation, spare time, marriage/partnership, relationships with children, oneself, friends/acquaintances, housing, and sexuality, as well as overall life satisfaction. Responses are provided on a 7-point scale, where 1 indicates “very dissatisfied” and 7 indicates “very satisfied.” Scores can be calculated for individual scales and as an indicator of overall life satisfaction, which, according to the instrument’s design, is the sum of the scores on seven scales: health, financial situation, spare time, oneself, friends and acquaintances, housing, and sexuality. The higher the score, the greater the life satisfaction in a given area. The reliability of the original version, determined by Cronbach’s alpha, is 0.95 for overall life satisfaction, and for individual scales, it ranges from 0.82 to 0.94. The Polish version of the questionnaire also has good psychometric properties; Cronbach’s alpha coefficients for individual scales range from 0.80 to 0.96. In a test–retest study with a one-month interval, correlation coefficients ranging from 0.61 (self) to 0.97 (relationship satisfaction) were obtained. Validity was assessed by comparing FLZ scores with the Satisfaction with Life Scale and the SF-36 questionnaire [18]. In the present analytic sample, Cronbach’s alpha coefficients ranged from 0.87 to 0.96 across the FLZ domains, and alpha for the overall seven-scale score was 0.94.

### 2.4. Statistical Analysis

The profiles were constructed using measurements of seven psychological indicators (psychological variables) obtained after weight loss. These comprised four IZZ subscales and three subscales from the Body Esteem Scale (specifically the female version). The overall IZZ score was excluded because it represents the sum of the four categories and would have resulted in double-weighting the same construct. All seven variables were standardised based on pre-clustering scores. First, a hierarchical cluster analysis was performed using Ward’s method with squared Euclidean distance, in accordance with standard cluster analysis principles [19]. Solutions ranging from k = 2 to k = 6 were compared using the average silhouette coefficient. The two-cluster solution yielded the highest silhouette value among the solutions examined (k = 2: 0.411; k = 3: 0.261; k = 4: 0.223; k = 5: 0.205; k = 6: 0.179). Subsequently, the two-cluster solution was validated using the k-means++ method with 100 random initialisations. Agreement between the Ward and k-means classifications was assessed using the Adjusted Rand Index (ARI), while cluster stability was further evaluated via bootstrapping with 1000 replications.

The variables used to define the clusters were summarised descriptively (means, standard deviations, standardised centroids, and Hedges’ g with 95% confidence intervals); *p*-values for the variables defining the profiles were not treated as an independent validation of the clusters. Differences in life satisfaction between profiles were assessed using Welch’s *t*-tests, reporting Hedges’ g with 95% confidence intervals. Mann–Whitney U tests were used as sensitivity analyses and led to the same substantive conclusions. The Holm correction was applied to the ten aspects of FLZ, while the overall FLZ score was analysed separately. The association between profile membership and four self-selected methods was assessed using the chi-square test of independence, reporting Cramer’s V with a bootstrapped 95% confidence interval. Analyses included only complete cases. No formal a priori power analysis was conducted, as the present study constitutes a secondary, exploratory cluster analysis. Sample adequacy was therefore assessed primarily based on the described internal stability analyses. The significance level was set at α = 0.05. Initial analyses were performed using Statistica 6.0; (StatSoft, Inc., Tulsa, OK, USA); additional cluster validation analyses and supplementary analyses were conducted during the revision process. These analyses were rechecked at the proof stage using Python 3.12.14 (Python Software Foundation, Beaverton, OR, USA), with NumPy 2.3.5, pandas 2.2.3, SciPy 1.17.0, and scikit-learn 1.8.0.

### 2.5. Ethical Issues

Participation was voluntary and anonymous. All participants were informed about the study purpose and procedure and provided informed consent before completing the anonymous questionnaires. Participants were informed that they could decline or discontinue participation without negative consequences. The broader project, including the continued use of the collected data, was approved by the Research Ethics Committee of the Faculty of Psychology, Kazimierz Wielki University in Bydgoszcz (protocol No. 1; approval date: 11 January 2024).

## 3. Results

### 3.1. Profile Extraction

Hierarchical cluster analysis using Ward’s method allowed for the evaluation of alternative clustering solutions; the mean silhouette coefficient was highest for k = 2 (0.411), compared to 0.261 for k = 3, 0.223 for k = 4, 0.205 for k = 5, and 0.179 for k = 6. The two-cluster classification obtained via Ward’s method was identical to the k-means++ solution (ARI = 1.00). Bootstrap analysis (1000 replications) indicated very high within-sample stability (median ARI = 1.00; approximately 95% of values fell within the 0.97–1.00 range). The first profile comprised 69 women and was characterised by relatively higher scores across four health behaviour categories and three body esteem dimensions; the second comprised 53 women and showed relatively lower scores. For clarity, the profiles were labelled as “relatively more favourable” and “relatively less favourable”; these labels are descriptive within the context of the study sample and do not represent clinical categories (Table 1). In the sensitivity analysis, the initial overall FLZ score did not differ between the groups that subsequently formed the two profiles (113.91 ± 32.80 vs. 114.34 ± 27.55; *p* = 0.938; Hedges’ g = −0.01). Of the seven initial profile indicators, only preventive behaviours and positive mental attitude differed significantly after Holm correction; both scores were initially lower in the group that subsequently formed the “relatively more favourable” profile.

### 3.2. Profiles and Life Satisfaction

Overall life satisfaction was higher in the relatively more favourable profile (245.75 ± 46.63) than in the relatively less favourable profile (197.77 ± 43.16), Welch’s t(115.84) = 5.88, *p* < 0.001, Hedges’ g = 1.06, 95% CI 0.68–1.44. All ten examined FLZ domains also differed after Holm correction (Table 2).

For the ten individual FLZ aspects, the reported *p*-values are adjusted using the Holm method; the overall FLZ score was analysed separately, and its *p*-value is not adjusted.

### 3.3. Profiles and the Method of Weight Reduction

Profile membership was associated with the self-selected weight loss method (χ^2^(3) = 53.80; *p* < 0.001; Cramer’s V = 0.66; bootstrapped 95% CI 0.54–0.79) (Table 3). The more favourable profile included 83.3% of women using dietary therapy and physical activity under the guidance of a dietitian and 93.8% of women using dietary therapy and physical activity on their own (Table 3). The less favourable profile included 78.6% of women using elimination diets and 75.0% of women using low-calorie diets. This pattern represents merely an observational co-occurrence. Since participants selected their own weight loss methods in a non-experimental study, the association does not allow for conclusions regarding effectiveness, causal superiority, or the independent contribution of physical activity; it may also reflect pre-existing differences in motivation, resources, support, or health behaviours.

## 4. Discussion

The study’s main finding is the heterogeneity observed among women who reached the same anthropometric endpoint. Women with a BMI within the normal range differed in their configuration of health behaviours and body-related evaluations, and the identified profiles were also associated with differences in life satisfaction. Thus, the results do not suggest that merely achieving a healthy BMI drives differences in psychosocial functioning; rather, they demonstrate that a similar final BMI can coexist with distinct behavioural and psychological patterns regarding the body [20,21,22].

Life satisfaction was used as an external variable because it was not included in the construction of the profiles. Women in the more favourable profile obtained higher overall FLZ scores and higher scores across all ten analysed life domains. These associations extend beyond the variables defining the profiles, but they should not be interpreted as evidence that the profile causes greater satisfaction in every area of life [23,24,25,26,27].

The profiles likely reflect broader configurations of characteristics than those included in the study. Sensitivity analyses of the baseline measurements revealed no differences in overall FLZ between the groups that subsequently formed the two profiles; following correction for multiple comparisons, the groups differed on only two of the seven profile indicators. This reduces—though does not eliminate—the possibility that the profiles observed after the reduction merely replicate pre-existing differences. The potential influence of other unmeasured factors—such as self-regulation, motivation, or social support—on both the choice of reduction method and the women’s subsequent functioning cannot be ruled out.

A more favourable profile was more common among women who reported strategies combining dietary intervention and physical activity. This association should be interpreted with caution. Physical activity was neither independently measured nor experimentally manipulated, and its contribution cannot be disentangled from dietary changes, motivation, support from a dietitian, prior weight-loss experiences, or other characteristics of the women choosing a given strategy [28,29,30,31,32,33,34,35].

Women who reported using elimination or low-calorie diets also achieved a BMI within the normal range, yet they were more likely to fall into the less favourable profile. This observation does not prove that these methods caused less favourable functioning or were less effective for weight loss; it merely indicates that different self-selected weight-loss methods co-occurred with different post-reduction profiles in this sample [36,37,38,39,40,41,42].

The results align with a biopsychosocial perspective, according to which BMI alone does not capture all significant aspects of functioning following weight loss. They support the inclusion of health behaviours, body image, and broader well-being—alongside anthropometric indicators—when describing the post-weight-loss state. However, current observational data do not allow for determining what form of further support, if any, would improve these outcomes [43,44,45].

The present study thus identifies descriptive profiles following weight loss and uses life satisfaction as an external variable associated with these profiles. The self-selected weight-loss method is a secondary descriptive characteristic, rather than an experimentally tested treatment or a basis for drawing conclusions regarding therapeutic efficacy [46,47,48,49].

### 4.1. Clinical Implications

The results suggest that assessing the functioning of individuals following weight loss could benefit from considering indicators beyond BMI, including health behaviours, body image, and life satisfaction. Such a broader assessment may help characterise the heterogeneity among women who have reached a similar anthropometric endpoint.

The present study did not test the effectiveness of specific follow-up programmes or isolate the effects of physical activity. Therefore, dietary support, psychological support, health education, or individually tailored physical activity should be regarded as potential directions for future prospective and interventional research rather than interventions demonstrated to be effective by the present data.

Likewise, profile membership should not be used as a clinical diagnostic category or as a direct indication for a specific form of care. Future studies should examine whether the exploratory profiles can be replicated and whether they prospectively predict needs, maintenance of weight loss, or response to particular forms of support.

### 4.2. Strengths and Limitations

A strength of the study is its focus on women who achieved the same pre-defined anthropometric endpoint and the use of validated tools covering health behaviours, body image, and multiple domains of life satisfaction. Life satisfaction was not used to generate the clusters, thereby serving as an external variable that characterised the identified profiles. The internal stability of the clusters was assessed using silhouette values, the agreement between Ward’s and k-means classifications, and bootstrapping.

The study was observational and non-experimental in nature, involving purposive sampling and self-selection of the weight-loss method. Consequently, the associations between the method and profile membership cannot be interpreted causally; they may reflect differences in motivation, resources, social support, prior health behaviours, or other unmeasured characteristics. Adherence to recommendations, strategy intensity, and changes in strategy during the weight-loss process were not systematically quantified.

The analytical sample was deliberately limited to women in a marriage or cohabiting relationship with at least one child, who had a BMI of 18.5–24.9 kg/m^2^, and who completed the second measurement. These criteria stemmed partly from the broader source project and limit the generalisability of the findings to other women, men, individuals who did not reach the target BMI, and those undergoing pharmacological or surgical treatment. Due to the study’s anonymous design and the lack of active individual monitoring following the initial measurement, it is not possible to reliably classify the reasons why the remaining initial participants did not return for the second measurement. Body weight and height were self-reported; therefore, the final BMI was not independently verified through anthropometric measurement. The second measurement was performed shortly after the reported achievement of the target BMI, so it is unknown whether the profiles were maintained over the long term. The profiles therefore describe only the 122 women who reported reaching the target BMI and completed the second assessment; their frequency among all initially assessed women or all women attempting weight loss cannot be estimated.

Cluster analysis is exploratory and depends on the selected variables. Although the two-profile solution showed strong internal stability in this sample, the sample size was modest, and the profiles require replication in independent and more diverse samples before they can be regarded as robust external categories. Baseline sensitivity analyses reduce concern that the profiles simply reflect broad pre-existing differences, but they do not establish causality.

Although the study did not control for socioeconomic status and income in this analysis, these factors may be associated with health behaviours, life satisfaction, and choice of weight loss strategies. Education-level data were available from the source project and were not linked to health behaviours, body image, general life satisfaction, or BMI. However, this does not replace a direct assessment of income or other socioeconomic factors. Future research should also incorporate objective anthropometric and physical activity measurements, longer follow-up periods, and a prospective assessment of social, psychological, and contextual factors.

## 5. Conclusions

Among women who achieved a BMI within the normal range after self-selected non-pharmacological weight loss, the same anthropometric endpoint coexisted with heterogeneous patterns of health behaviours and body esteem.

The two exploratory profiles were associated with differences in overall life satisfaction and its examined domains, indicating that BMI alone does not capture the full range of post-weight-loss functioning in this specific sample.

Self-selected weight-loss strategies were distributed differently across the profiles, but this observational association does not demonstrate effectiveness, causal advantage, or an independent effect of physical activity. The profiles require replication in independent and more diverse samples before clinical application can be considered.

## Figures and Tables

**Table 1:** Descriptive characteristics of the health behaviour and body esteem variables used to define the profiles.

Variable	More Favourable Profile M ± SD N = 69	Less Favourable Profile M ± SD N = 53	Standardized Centroid More/Less	Hedges’ g (95% CI)
Proper eating habits	25.70 ± 2.47	16.83 ± 3.42	0.73/−0.95	3.02 (2.50–3.54)
Preventive behaviours	24.17 ± 3.48	17.04 ± 3.95	0.61/−0.79	1.92 (1.49–2.35)
Positive mental attitude	24.77 ± 3.01	19.47 ± 3.82	0.54/−0.70	1.56 (1.15–1.96)
Health practices	24.55 ± 2.97	17.66 ± 3.33	0.65/−0.84	2.19 (1.74–2.64)
Sexual attractiveness	55.30 ± 5.36	45.57 ± 5.05	0.60/−0.78	1.85 (1.43–2.28)
Weight concern	43.04 ± 3.93	33.13 ± 5.49	0.64/−0.83	2.11 (1.66–2.55)
Physical condition	39.20 ± 3.67	33.28 ± 4.46	0.52/−0.68	1.46 (1.06–1.86)

**Table 2:** Differences in life satisfaction between profiles, with effect sizes and *p*-values.

Aspect of Life Satisfaction	More Favourable Profile M ± SD N = 69	Less Favourable Profile M ± SD N = 53	*p*-Value	Hedges’ g (95% CI)
General score FLZ (7 scales)	245.75 ± 46.63	197.77 ± 43.16	<0.001	1.06 (0.68–1.44)
Health	37.57 ± 9.39	31.83 ± 9.38	0.001	0.61 (0.24–0.97)
Work and profession	31.58 ± 8.04	25.98 ± 7.47	<0.001	0.71 (0.35–1.08)
Financial situation	32.88 ± 8.50	26.83 ± 7.14	<0.001	0.76 (0.39–1.13)
Spare time	35.88 ± 7.40	28.89 ± 8.19	<0.001	0.90 (0.52–1.27)
Relationships with one’s own children	35.87 ± 7.65	27.72 ± 7.57	<0.001	1.06 (0.68–1.44)
Oneself	35.97 ± 8.56	27.34 ± 8.44	<0.001	1.01 (0.63–1.39)
Friends and acquaintances	32.32 ± 6.34	26.02 ± 6.87	<0.001	0.95 (0.58–1.33)
Accommodation	32.72 ± 6.50	26.32 ± 6.40	<0.001	0.99 (0.61–1.36)
Marriage or relationship	34.55 ± 7.90	28.75 ± 6.28	<0.001	0.80 (0.43–1.17)
Sexuality	38.41 ± 7.43	30.55 ± 6.70	<0.001	1.10 (0.71–1.48)

Note: The overall FLZ score comprises seven scales: health, financial situation, leisure time, oneself, friends and acquaintances, housing, and sexuality.

**Table 3:** Distribution of weight reduction methods in the selected profiles (number and percentage structure).

Weight Loss Method	More Favourable Profile *n* (%)	Less Favourable Profile *n* (%)	Total *n*
Diet therapy and physical activity under the supervision of a dietitian	25 (83.3%)	5 (16.7%)	30
Independent diet therapy and physical activity	30 (93.8%)	2 (6.2%)	32
Elimination diets	6 (21.4%)	22 (78.6%)	28
Low-calorie diets	8 (25.0%)	24 (75.0%)	32

## Data Availability

Data are not publicly available due to participant consent and privacy concerns. They can be made available by the corresponding author upon reasonable request, provided applicable regulations and ethical documentation allow.
